# The Role of Noncoding RNAs in Double-Strand Break Repair

**DOI:** 10.3389/fpls.2019.01155

**Published:** 2019-09-27

**Authors:** Nathalie Durut, Ortrun Mittelsten Scheid

**Affiliations:** Gregor Mendel Institute of Molecular Plant Biology, Austrian Academy of Sciences, Vienna BioCenter (VBC), Vienna, Austria

**Keywords:** double-strand break, DNA repair, noncoding RNAs, CRISPR/Cas, plants

## Abstract

Genome stability is constantly threatened by DNA lesions generated by different environmental factors as well as endogenous processes. If not properly and timely repaired, damaged DNA can lead to mutations or chromosomal rearrangements, well-known reasons for genetic diseases or cancer in mammals, or growth abnormalities and/or sterility in plants. To prevent deleterious consequences of DNA damage, a sophisticated system termed DNA damage response (DDR) detects DNA lesions and initiates DNA repair processes. In addition to many well-studied canonical proteins involved in this process, noncoding RNA (ncRNA) molecules have recently been discovered as important regulators of the DDR pathway, extending the broad functional repertoire of ncRNAs to the maintenance of genome stability. These ncRNAs are mainly connected with double-strand breaks (DSBs), the most dangerous type of DNA lesions. The possibility to intentionally generate site-specific DSBs in the genome with endonucleases constitutes a powerful tool to study, *in vivo*, how DSBs are processed and how ncRNAs participate in this crucial event. In this review, we will summarize studies reporting the different roles of ncRNAs in DSB repair and discuss how genome editing approaches, especially CRISPR/Cas systems, can assist DNA repair studies. We will summarize knowledge concerning the functional significance of ncRNAs in DNA repair and their contribution to genome stability and integrity, with a focus on plants.

## Introduction

Preservation of genome integrity is a prerequisite for proper cell function and faithful transmission of the genome to progeny. However, genome stability and integrity are constantly challenged by various endogenous (metabolic products, radicals, stalled replication forks) and exogenous (energetic radiation, chemical pollutants) factors that cause different kinds of DNA lesions (Mehta and Haber, 2014). To protect their genome, eukaryotes have evolved a sophisticated and highly coordinated network to recognize, signal, and repair DNA lesions, summarized as the DNA damage response (DDR) (Ciccia and Elledge, 2010; Manova and Gruszka, 2015). The risk to genome integrity from DNA damage differs depending on the type of lesion. While damage affecting only one strand of the DNA molecule can be corrected by intact information on the opposite strand, breaks in both DNA strands (double-strand breaks [DSBs]) are more difficult to process and present the risk of losing genetic information upon repair, especially if they occur in germinal cells in animals or in the pool of plant cells that form the gametes (Waterworth et al., 2011; Helleday et al., 2014; Gaillard et al., 2015; Ghosh et al., 2018). Two major pathways safeguard the genome from DSB’s deleterious consequences: the error-prone pathway called nonhomologous end-joining (NHEJ) and the more faithful pathway called homologous recombination (HR). Many studies have been conducted to understand DSB repair mechanisms leading to the identification of numerous proteins and the genes encoding them (Mehta and Haber, 2014; Manova and Gruszka, 2015). Recently, ncRNAs have also been reported to be involved in DNA repair, adding another unexpected role of ncRNAs to their growing spectrum of biological functions (Hawley et al., 2017; Michelini et al., 2018; Thapar, 2018).

Double-strand breaks arise from high-energy radiation, exposure to reactive oxygen species, cross linkers, radiomimetic drugs, or spontaneously during DNA replication and transposon or transgene integration. DNA lesions are usually randomly generated within the genome, except when they are induced during meiosis to generate genetic variations (Mehta and Haber, 2014). Temporal and site-specific induction of DSBs was used repeatedly in research to study molecular repair mechanisms, applying restriction enzymes, zinc finger nucleases, transcription activator-like effector nucleases (TALENs), or clustered regularly interspaced short palindromic repeats and the associated endonuclease Cas9 (CRIPSR/Cas9) (Doudna and Charpentier, 2014; Chandrasegaran and Carroll, 2016; Weeks et al., 2016). Due to its high specificity, simplicity of cloning, and diversity of applications, CRISPR/Cas systems surpass the other options. They represent promising tools for studying the role of ncRNAs in DNA repair and can be easily introduced into different plant species. As most studies have been done with mammalian systems, this review summarizes the major mechanisms of DSB repair and the involvement of ncRNAs in response to DNA damage in plants. We will also discuss how biotechnological applications of CRISPR/Cas can assist DNA repair studies.

## DNA Damage Response and DSB Repair

DNA repair requires a plethora of enzymes and proteins that work together to ensure efficient and rapid repair of different kinds of lesions. It is coordinated by the DDR, a highly conserved pathway in animals, fungi, and plants that ensures faithful transmission of genetic information to subsequent generations. Failures in DDR lead to malignant transformation, cell death, retardation of growth and development, or sterility (Jackson and Bartek, 2009; Ciccia and Elledge, 2010; Yoshiyama et al., 2013). The DDR pathway is initiated with the activation of two phosphatidylinositol 3 kinase-like protein kinases: ATM (ataxia telangiectasia mutated) and ATR (Rad3-related) (Kurz and Lees-Miller, 2004; Shechter et al., 2004). In *Arabidopsis thaliana*, so far the best studied model plant for DDR, ATM is activated in response to DSBs, while ATR is involved in a wide range of DNA lesions, especially those associated with DNA replication defects (Garcia et al., 2003; Culligan et al., 2004; Culligan et al., 2006). These kinases initiate a signaling cascade to phosphorylate various proteins, which, in turn, amplify the signal by recruiting ATM/ATR substrate and inducing cell cycle arrest and DNA repair (Ciccia and Elledge, 2010). Phosphorylation of the histone variant H2A.X is among the earliest events occurring during DDR, leading to the formation of ɣH2A.X foci that act as an amplification signal of DNA damage to recruit chromatin remodeler complexes and additional DNA repair factors that accumulate in foci (Paull et al., 2000; Friesner et al., 2005; Fillingham et al., 2006). In parallel, the cell cycle is arrested to provide time for DNA repair prior to replication. The plant-specific transcription factor SOG1 (suppressor of gamma response 1), regulated by ATM phosphorylation, participates in the control of this process (Yoshiyama et al., 2009; Bourbousse et al., 2018; Ogita et al., 2018). If DNA is irreparably damaged, programmed cell death or endoreduplication, a modified version of the cell cycle without cell division leading to the formation of polyploid cells, is initiated (Fulcher and Sablowski, 2009; Adachi et al., 2011; Yoshiyama et al., 2013).

Among the different types of DNA lesions, DSBs represent the most deleterious. They are corrected by two main pathways conserved between eukaryotes and prokaryotes: NHEJ and HR. Nonhomologous end-joining is a fast process that operates in any phase of the cell cycle and simply ligates the two ends of a DSB, with no potential for restoring the original DNA sequence (Mladenov and Iliakis, 2011; Waterworth et al., 2011). Double-strand break sites are recognized by the conserved Ku70/Ku80 heterodimer complex (Walker et al., 2001) that recruits DNA-dependent protein kinases (DNA-PKcs) to stabilize DNA ends. DNA ends are then prepared for ligation by removing or filling overhanging-ends, and blunted-ends are ligated, in mammals by a complex of XLF (XRCC4-like factor), XRCC4 (X-ray repair cross-complementing protein 4), and DNA ligase IV (West et al., 2000; Lee et al., 2003; Ahnesorg et al., 2006). In *A. thaliana*, disruption of *Ku70*, *Ku80*, or *DNA ligase IV* genes increases sensitivity to DSB-inducing agents, and *KU80* inactivation reduces NHEJ repair efficiency (Tamura et al., 2002; West et al., 2002; Friesner and Britt, 2003). The function of the Ku70/Ku80 complex for NHEJ has also been confirmed in crops such as rice, wheat, and barley, suggesting that it is well conserved across evolution (Nishizawa-Yokoi et al., 2012; Gu et al., 2014).

Restoration of the initial sequence at the DSB lesion by HR depends on the presence of an intact copy and is therefore more frequent after the S phase and during G2 of the cell cycle. In somatic plant cells, it represents a minor DSB repair pathway, but is highly important during meiosis (Puchta, 2005; Vu et al., 2014; Melamed-Bessudo et al., 2016; Weimer et al., 2016). Preceding HR, DSBs are detected by the MRN complex consisting of MRE11 (meiotic recombination 11), RAD50 (radiation sensitive 50), and NBS1 (Nijmegen breakage syndrome 1), which activates ATM and/or ATR by autophosphorylation (Uziel et al., 2003; Williams et al., 2007). This is followed by rapid phosphorylation of various DNA repair factors such as H2A.X, BRCA1 (breast cancer 1), and EXO1 (exonuclease 1) (Cortez et al., 1999; Burma et al., 2001; Matsuoka et al., 2007; Bolderson et al., 2010). Nuclease and helicase activities of BRCA1, EXO1, and MRE11 initiate end resection, resulting in single-strand 3′ overhangs that are rapidly coated by RPA (replication protein A), which is subsequently replaced by RAD51 (radiation sensitive 51) in the presence of BRCA2 (breast cancer 2) (Ciccia and Elledge, 2010). The nucleoprotein filament invades a homologous template, and DNA synthesis is carried out by either single-strand annealing, synthesis-dependent strand annealing (SDSA), or double-strand break repair (mechanistic details in Filippo et al., 2008; Gill et al., 2015). Plants have orthologs of the MRN complex (Gallego et al., 2001; Akutsu et al., 2007). As in animals, AtRAD50 and AtMRE11 proteins interact with one another (Daoudal-Cotterell et al., 2002), as do MRE11 and NBS1 in *Arabidopsis*, rice and maize (Akutsu et al., 2007; Waterworth et al., 2007). Mutations in *AtRAD50* and *AtMRE11* render plants hypersensitive to DSBs and sterile, suggesting a role for the MRN complex in HR during meiosis (Gallego et al., 2001; Bundock and Hooykaas, 2002; Puizina et al., 2004).

Backup pathways independent of KU and DNA ligase IV components have also been described in mammals and plants, including microhomology-mediated end joining and alternative NHEJ (a-NHEJ) (Bennardo et al., 2008; Charbonnel et al., 2011; Jia et al., 2013). These repair pathways rely on microhomologies between sequences flanking the DSBs. The frequency and efficiency of all repair mechanisms combined determine the balance between genome stability and the production of genetic diversity.

## Noncoding RNAs in DNA Repair

Noncoding RNAs represent the vast majority of transcripts from the nuclear genome in eukaryotes (Yamada et al., 2003; Carninci et al., 2005; David et al., 2006; Li et al., 2006; Marchese et al., 2017). In addition to growing evidence for their multifunctional role in diverse biological processes (Gomes et al., 2013; Borges and Martienssen, 2015; Liu et al., 2015), a connection between ncRNAs and DNA repair in genome safeguarding has been recently described (Francia, 2015; Thapar, 2018). Noncoding RNAs have been proposed to promote DNA damage signaling and repair from damaged loci by (1) regulating the abundance of DNA repair proteins, (2) guiding DNA homology-directed repair, or (3) providing an intact copy used as a template for DSB repair (Yang and Qi, 2015; Khanduja et al., 2016). These potential functions of ncRNAs are discussed in the following section.

### Role of Small ncRNAs in DNA Repair

Noncoding RNAs comprised several classes, differing in both length and function (Hombach and Kretz, 2016). Besides the well-established role of small ncRNAs (sncRNAs) (<200 nt) in epigenetic regulation (Chen and Xue, 2016), several studies report their participation in the DDR pathway (Chowdhury et al., 2013; Wan et al., 2014). Small ncRNAs belonging to the miRNA subclass modulate the expression of central components of the DSB repair machinery such as ATM, H2A.X, and BRCA1, which in turn can modulate the expression of miRNAs either at the transcriptional or posttranscriptional level. Mutations in genes coding for DICERs (proteins that process sRNAs from double-stranded precursors) or AGOs (argonaute proteins that bind sRNAs) affect DNA repair efficiency, suggesting the importance of miRNAs in DNA repair (Wang and Taniguchi, 2013). As the involvement of miRNAs in DDR is described in excellent reviews (Chowdhury et al., 2013; Sharma and Misteli, 2013; Wan et al., 2014), here we will rather describe sncRNAs directly induced by DNA damage and connected with the site of the lesions.

Using transgenic *Arabidopsis* plants, expressing the *I-SceI* endonuclease and a β-glucuronidase (GUS)–based recombination reporter construct that contains the *I-SceI* site, Wei et al. (2012) reported that 21 nt RNAs are induced in response to DSBs. These DSB-induced small RNAs (diRNAs) are specifically produced from sequences surrounding the DSB site and are generated from sense and antisense transcription. Their biogenesis depends on ATR, RNA polymerase IV, RDR (RNA-dependent RNA polymerase), and DICER proteins in *Arabidopsis*. Their depletion in *dicer* mutants affects neither the formation of ɣH2A.X nor DDR foci, suggesting that they act downstream or in parallel to the recruitment of DNA repair factors. Moreover, their depletion does not alter the level of HR gene expression, indicating that they act differently from canonical miRNAs. Double-strand break-induced small RNAs assemble with AGO2, a central factor in the RNA-induced silencing complex (RISC). In mammalian cells, reduced amounts of DICER or AGO2 impair DSB repair (Wei et al., 2012), and interaction between diRNAs and AGO2 is essential for initiation of HR *via* recruitment of RAD51 protein, possibly by guiding the diRNA/AGO2/RAD51 complex to the lesion through base pairing with sequences flanking the DSB (Gao et al., 2014). To better characterize the function of these diRNAs, Miki et al. (2017) used CRISPR/Cas9 and TALEN technologies to generate site-specific DSBs in *Arabidopsis* and rice genomes. Double-strand breaks were introduced within GUS transgenes or in endogenous loci. While no diRNAs were observed at the tested endogenous loci, they strongly accumulated at transcribed transgenes, independently of whether the transgene was constructed as an HR reporter or not. This suggests that diRNA formation depends on transcription but not on HR repair. As *dcl2*, *dcl4*, and *rdr6* mutants impair diRNA accumulation, the RdDM pathway contributes to their production, but DSB induction and RNA polymerase II transcription are also needed at the damaged site.

Mammalian cells have a similar system: DICER and DROSHA, which produce double-stranded sRNAs in the RNA interference (RNAi) pathway, are required for foci formation and cell cycle control during DDR response. They produce DICER- and DROSHA-dependent 21-nt small RNAs, named DDRNAs (DDR RNAs), from the site of the lesion. Their production depends on a functional MRN complex (Francia et al., 2012). RNAse A treatment of damaged cells reduces the formation of DDR foci, which can be restored by adding synthesized RNAs or total RNA from damaged cells. Precursors of DDRNAs are produced by RNA polymerase II (Pol II), as total RNA from cells treated with the transcription inhibitor α-amanitin prior to DNA damage does not restore DDR foci formation. DDRNAs are specifically localized at sites homologous to the damaged site and seem to be derived from precursors called damage-induced long-noncoding RNAs (dilncRNAs) transcribed by RNA pol II in a bidirectional manner from DSB ends (Michelini et al., 2017). Damage-induced long-noncoding RNAs interact with DDRNAs and specifically guide them to the damaged site through base-pairing. This pairing is essential for DDR foci formation as antisense oligonucleotides and RNA pol II transcription impair their formation.

The induction of sncRNAs upon DNA damage seems to be an evolutionary conserved process. In the fungus *Neurospora crassa*, DNA lesions induce expression of an AGO protein (QDE2) and of 20 to 21 nt sncRNAs named QDE2-interacting RNAs (qiRNAs), generated by the RNA-dependent RNA polymerase QDE1 (Lee et al., 2009). Although their role in DDR remains unclear, mutating proteins involved in their biogenesis results in DNA damage sensitivity. In *Drosophila melanogaster* cells, linearized DNA plasmids that mimic DSB ends induce the formation of 21 nt sncRNAs named endo-siRNAs, *via* the activity of the Dicer-2 protein Dcr-2 (Michalik et al., 2012). Transcription occurring within the vicinity of broken DNA sites amplifies the small RNA response, showing that endogenous mRNA can serve as a precursor for sncRNA generation. However, general perturbation of siRNA biogenesis in *Drosophila* cell culture does not affect DNA repair efficiency; thus, a connection with DDR is not yet clear (Schmidts et al., 2016).

Noncoding RNAs are also involved in the repair of DNA lesions other than DSBs. In *Arabidopsis*, distortion of the DNA helix by the formation of DNA photoproducts caused by UV irradiation induces 21 nt sRNAs, which participate in the DNA repair recognition process (Schalk et al., 2017). These sRNAs are transcribed by RNA polymerase IV, RDR2 (RNA-dependent RNA polymerase) and processed by DICER4. Similar to the case for DSBs, several RdDM components are necessary for their production, and mutation of the RdDM components reduces UV-induced DNA repair. They appear to act together with AGO1 and the DNA damage-binding protein 2, likely also by providing RNA/DNA complementarity at the damaged site.

**Figure 1:**
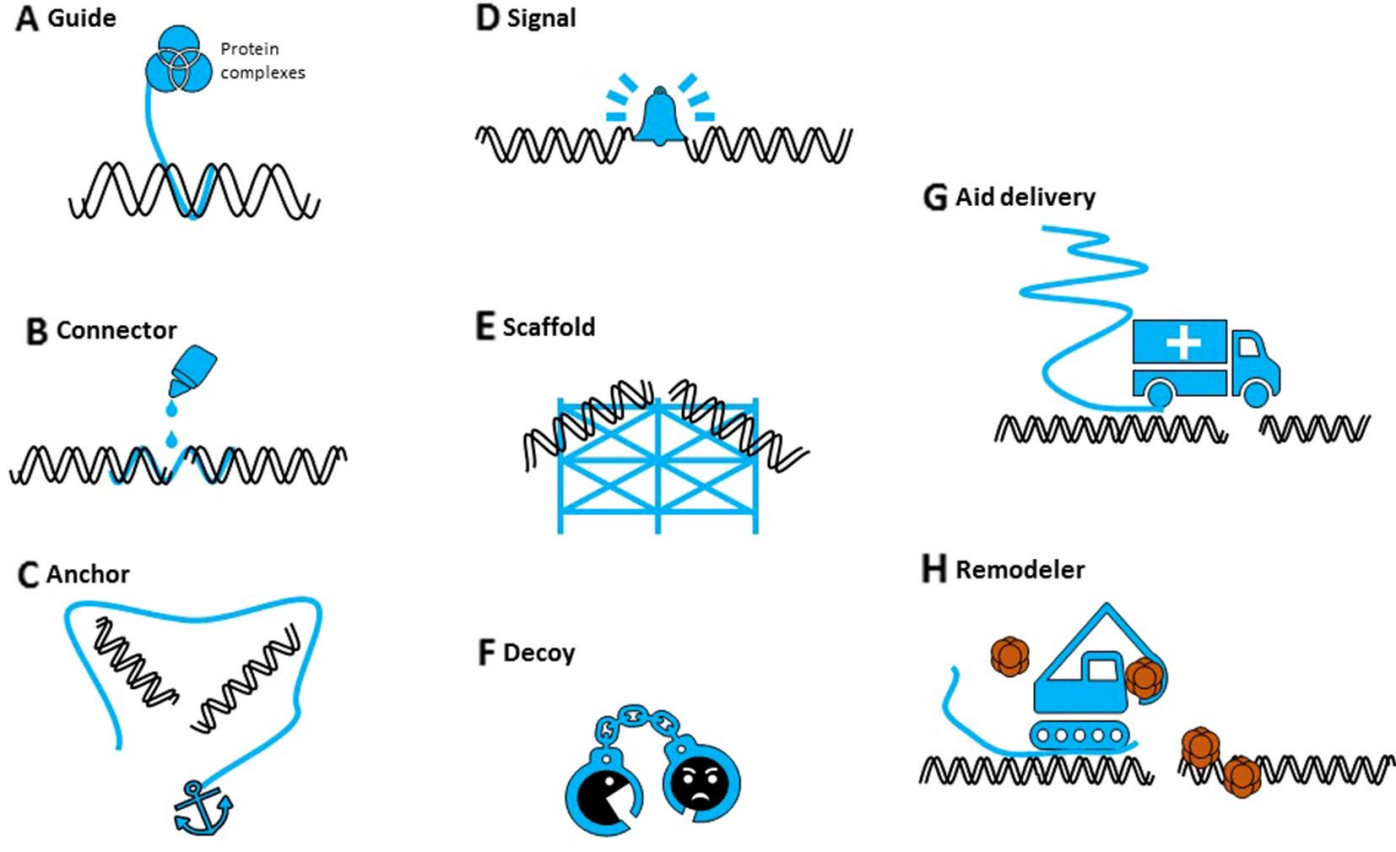
The different potential roles of noncoding RNAs in DNA repair. Noncoding RNAs (in blue) can **(A)** guide proteins to specific sites in the genome, **(B)** potentially hold broken ends together (connector), **(C)** mediate DNA-DNA or DNA-protein interactions (anchor), **(D)** serve as an indicator (signal) of DNA damage, **(E)** provide a repair scaffold, **(F)** keep interfering components away (decoy), **(G)** recruit repair proteins (aid delivery), or **(H)** initiate chromatin reconstruction (remodeler).

Potential mechanisms by which sncRNAs could participate in DNA repair have been proposed (**Figure 1**): degrade nascent RNA from damaged site, recruit chromatin remodeling complexes to modify chromatin structure, recruit/guide DDR factors to DSB site, or serve as template for DNA synthesis (Chowdhury et al., 2013). Despite the different names and nomenclature, sncRNAs are an evolutionary conserved component of DDR, although their precise role during DNA repair mechanisms, commonality, and specificity remains to be investigated. Other persisting questions are those for the origin of their precursors, their (local) processing, if they form complexes with repair proteins, their stability, and their fate after repair.

### Role of lncRNAs in DNA Repair

As described, short RNAs are mostly derived from longer, usually double-stranded precursors, by Dicer proteins. However, it recently became apparent that longer noncoding RNAs (lncRNAs) are also involved in DDR and DSB repair, without being processed into shorter products. The first lncRNA identified to be transcribed in response to DNA damage was *ncRNA* *_CCND1_* derived from the 5′ regulatory region of the *CCND1* (cyclin D1) gene in HeLa cells (Wang et al., 2008). It interacts with the RNA-binding protein TLS (translocated in liposarcoma) to repress the *CCND1* gene, which encodes a cell cycle regulator, by inhibiting the activity of the histone acetyltransferase complex p300/CBP. An enhancer region regulating the *CCND1* gene is also the site of sense and antisense transcription, producing two lncRNAs *CUPID1* and *CUPID2* that might influence the choice of the repair pathway (Betts et al., 2017). Other mammalian lncRNAs induced upon genotoxic stresses are *lincRNA p21* (long intergenic ncRNA p21) and *PANDA* (p21-associated ncRNA DNA damage-activated), both of which are transcribed in an antisense manner upstream of the *CDKN1A* (cyclin-dependent kinase inhibitor 1A) gene and modulate the expression of apoptotic genes upon DNA damage (Huarte et al., 2010; Hung et al., 2011). *lincRNA p21* interacts with hnRNA-K (heterogeneous nuclear ribonucleoprotein K), repressing the expression of *p53*, which encodes the tumor suppressor transcription factor p53. *PANDA* negatively regulates apoptosis by interacting with the transcription factor NF-YA (nuclear factor Y). Another example is the lncRNA *DINO* (damage-induced noncoding), which is also transcribed upstream of the *CDKN1A* gene in a p53 dependent-manner. It amplifies the DDR by stabilizing the p53 protein to promote p53-dependent gene expression, cell cycle arrest, and apoptosis (Schmitt et al., 2016). The lncRNA *GUARDIN* is also a p53-responsive lncRNA playing a key role in genome stability in response to genotoxic stress (Hu et al., 2018). It acts as a decoy to sequester *miRNA-23a* and maintain the expression of *TRF2* (telomeric repeat factor 2) to prevent chromosome end-to-end fusion. Moreover, it regulates the stability of BRCA1 and favors its heterodimerization with BARD1 (BRCA1-associated RING domain protein 1). An lncRNA termed *DDSR1* (DNA damage-sensitive RNA1) is also induced in response to DNA damage in an ATM-dependent manner and controlled by the NF-κB transcription factor (nuclear factor “kappa-light-chain enhancer” of activated B cells) (Sharma et al., 2015). It acts as a tumor suppressor by regulating DNA end resection. It modulates the access of BRCA1 to the damaged site by sequestering the BRCA1-RAP80 complex as well as interacting with hnRNPUL1 (heterogeneous nuclear ribonucleoprotein U-like 1), which promotes DNA end resection (Polo et al., 2012). Loss of *DDSR1* impairs DDR signaling and reduces DNA repair (Sharma et al., 2015). The lncRNA prostate cancer–associated transcript 1 (*PCAT-1*) reduces HR *via* down-regulation of BRCA2, by binding to the 3′ untranslated region of *BRCA2* and down-regulating the stability of its mRNA *via* posttranscriptional modifications (Prensner et al., 2014). The lncRNA gene for apoptosis and differentiation in epithelia (*JADE*) is induced upon DNA damage in an ATM-dependent manner and is transcribed in the antisense direction to *JADE1* (Wan et al., 2013). *JADE* promotes *JADE1* expression by interacting with BRCA1 to recruit the transcriptional coactivator P300. *JADE1* encodes a component of the H4 histone acetylation complex (HAT HBO1, human acetylase binding to ORC1), associated with transcriptional activation upon DDR. TODRA (transcribed in opposite direction of RAD51) is an antisense lncRNA transcribed 69 bp upstream of the *RAD51* gene (Gazy et al., 2015). It promotes HR in an RAD51-dependent manner by regulating RAD51 activity and expression. For other *trans*-acting lncRNAs connected with DNA repair functions in mammals, we refer to the following reviews (Wu and Wang, 2017; Thapar, 2018). Long-noncoding RNAs that act in *cis*, directly at the lesion site, have also been reported (D’Alessandro et al., 2018). Named dilncRNAs (for damage-induced lncRNAs), they are transcribed in both directions from the damaged site. They form DNA : RNA hybrids and recruit the repair factors BRCA1, BRCA2, and RAD51, which are involved in HR. Transfecting cells with oligonucleotides complementary to these dilncRNAs reduces HR efficiency.

All of the aforementioned studies were conducted in animal cells. Despite numerous and important examples of the function of lncRNAs in plants (Liu et al., 2015), a functional involvement in DDR has not yet been documented. Only one study reported that a small fraction of transposable elements and lncRNAs were expressed after DSB damage in *Arabidopsis* (Wang et al., 2016). The authors investigated the transcriptome of WT and *atm* mutant plants after DNA damage and reported that 0.6% of TEs and lncRNAs are induced, and the expression of greater than 95% of them is ATM-dependent. They showed that protein-coding genes localized in the proximity of damage-induced TEs or lncRNAs are frequently coexpressed, suggesting that the TEs and lncRNAs act as controlling elements of these neighbors.

As their small counterparts, lncRNAs might act as signals in DDR, scaffolds for DDR factors or chromatin remodeling complexes, decoys to keep off interfering molecules, or regulators of DDR gene expression (**Figure 1**) (Chowdhury et al., 2013; Sharma and Misteli, 2013). They could contribute to the regulation of DNA damage repair in multiple ways, at the lesion site itself or indirectly, by their own transcription or by interaction with proteins or DNA. It is now clear that we should consider both small and long ncRNAs as integral components of DDR, although their individual roles and mechanisms of action need to be revealed.

## RNA as a Template for DNA Repair

While the transfer of genetic information from DNA to RNA is the default path of gene expression, the reverse direction from RNA to DNA is an integral part of the life cycle of retroviruses, retrotransposons, and during telomere synthesis (Baltimore, 1985; Autexier and Lue, 2006; Sztuba-Solinska et al., 2011). This raises the possibility that RNA templates can represent faithful copies for accurate DNA repair. Several studies have demonstrated that artificial as well as endogenous RNAs can drive homology-directed recombination in both prokaryotic and eukaryotic cells. A direct exchange of genetic information between RNA and DNA molecules was first shown in the yeast *Saccharomyces cerevisiae* where artificial RNA oligonucleotides complementary to DSB ends were able to drive HR-mediated DNA repair (Storici et al., 2007). Similar results have since been observed in bacteria, human cells, and the ciliate *Oxytricha trifallax* (Nowacki et al., 2008; Shen et al., 2011). These findings challenged the conventional view that HR occurs only between two DNA molecules and indicated that RNA can guide genomic rearrangements and modifications. Later, with a robust genetic reporter system to directly detect HR events, initiated by RNA molecules in *cis* or *trans*, it was demonstrated that endogenous RNAs can mediate HR with chromosomal DNA in *S. cerevisiae* in a RAD52 dependent-manner (Keskin et al., 2014; Keskin et al., 2016). RAD52 promotes RNA-DNA annealing *in vitro* and stimulates RNA-directed HR repair through RNA-DNA complementarity especially in the absence of DNA templates (McDevitt et al., 2018). In mammalian cells, nascent RNAs associate with NHEJ factors to promote DSB repair in actively transcribed regions (Chakraborty et al., 2016). The authors showed that, after DSB induction, several NHEJ repair factors associate with nascent RNA molecules and RNA polymerase II in actively transcribed regions and that transcription is necessary for NHEJ recruitment. Depletion of NHEJ factors impairs DSB repair only in transcribed regions, suggesting that inactive regions might be repaired by a-NHEJ pathways.

Again, corresponding evidence from plants is missing. RNA-templated DNA repair has been postulated to explain some observations of non-Mendelian inheritance, but this hypothesis still awaits independent validation (Lolle et al., 2005; Xu et al., 2007; Miyagawa et al., 2013).

Fortunately, molecular techniques for RNA analyses in general, and lncRNA especially, have made tremendous progress, enabling researchers to sequence RNA from rare, limited material, with high resolution and coverage, and with a more direct representation of variants, intermediates, and modifications (Lagarde et al., 2017; Uszczynska-Ratajczak et al., 2018; Deshpande et al., 2019). Moreover, the visualization of rare RNAs using cytological techniques is becoming possible (Duncan et al., 2016). These techniques are complementing the conventional functional studies of ncRNAs currently performed with overexpressing lines or functional elimination by RNA interference, antisense oligonucleotides (ASO), or mutations. The latter toolbox has now also been substantially extended by the development of programmable, site-specific nucleases. They allow the study of DDR to become independent of random mutation events and have the potential to induce DSBs at almost any desired site within the genome and, in contrast to RNA interference techniques, can also be applied to target nuclear RNAs. Their potential applications to the study of the role of ncRNAs in the context of DNA repair are described below.

## Application of CRISPR/Cas Technology to Study ncRNAs in DNA Repair

The CRISPR/Cas endonuclease system was rapidly adopted and optimized in many experimental systems to mediate genome editing. It was preferred over other endonucleases due to its high specificity, simplicity, versatility, and diversity of applications. In standard applications for mutagenesis, the system needs two components: the CRISPR-associated endonuclease (Cas9) and a single gRNA (sgRNA) containing a sequence complementary to the genomic target site, which must be located next to a 3- to 6-nt-long protospacer adjacent motif (PAM), as well as an RNA sequence that associates with the Cas9 protein (Jinek et al., 2012). Cas9, recruited to the target region, cleaves the DNA producing a DSB. Distal mismatches next to the PAM site can lead to off-targets at undesired sites, but so far, only a few studies have reported off-target mutagenesis in plants (Xie et al., 2014).

Like randomly occurring DSBs, CRISPR/Cas9-induced DSBs are repaired either by HR or NHEJ, with more or less fidelity and different outcomes. NHEJ-mediated repair is suitable for generating gene knockout mutations as it often leads to small deletions, frame shifts, or modified binding sites, while HR is more applicable to achieve DNA knock-in or gene replacement (i.e. gene targeting) in the presence of a donor template (Kim and Kim, 2014). CRISPR/Cas9 mutagenesis has been successfully applied in many organisms including plants (Kumlehn et al., 2018). Its targeting efficiency and capacity have been rapidly improved by optimizing and specifying Cas9 expression with cell- and tissue-specific promoters (Hyun et al., 2015; Wang et al., 2015; Yan et al., 2015; Mao et al., 2016; Miki et al., 2018; Wolter et al., 2018). Gene targeting remains more challenging, but the number of successes in engineering new or fine-tuning existing traits in crop plants is rapidly increasing (Hyun et al., 2015; Wang et al., 2015; Yan et al., 2015; Mao et al., 2016; Sun et al., 2016; Begemann et al., 2017; Dahan-Meir et al., 2018; Li et al., 2018; Miki et al., 2018; Wolter et al., 2018). This is in part due to the application of Cas variants that produce nicks (single-strand breaks) rather than DSBs, or the use of Cas12 that induces DSBs further away from the PAM sequence, targets AT-rich regions better, and produces 5′ overhangs that likely promote HR (Schindele et al., 2018). Moreover, many more structural and functional variations of CRISPR/Cas systems have been identified (Shmakov et al., 2017), and the ease, robustness, and flexibility of this technology make them attractive and versatile components to introduce different types of DNA lesions in plants. Using one protein but two or multiple sgRNAs produces simultaneous cuts at multiple sites to create larger deletions after repair (Zhou et al., 2014; Xie et al., 2015; Lowder et al., 2017; Pauwels et al., 2018). Therefore, Cas variants with different endonuclease activities, tethered to specific genomic sites by their respective gRNAs, in specific cell types, and at chosen times, represent great tools for studying DNA repair processes in plants. The CRISPR/Cas13 ribonuclease family, which accepts only RNA molecules as a substrate (Abudayyeh et al., 2017), adds another valuable option for modifying polynucleotides with high specificity. Both DNA and RNA sequences can be further modified by base editing, an alternative mutagenic mechanism triggering special repair pathways. Variations of the Cas protein with mutations in its two nuclease domains render the protein catalytically inactive (deadCas or dCas), but it maintains its ability to associate with the gRNA, converting it into a DNA-binding protein (Qi et al., 2013). Fused to a cysteine or adenine deaminase domain, the complex can drive precise point mutations by base editing C/G to T/A or T/A to C/G, respectively (Komor et al., 2016; Cox et al., 2017).

**Figure 2:**
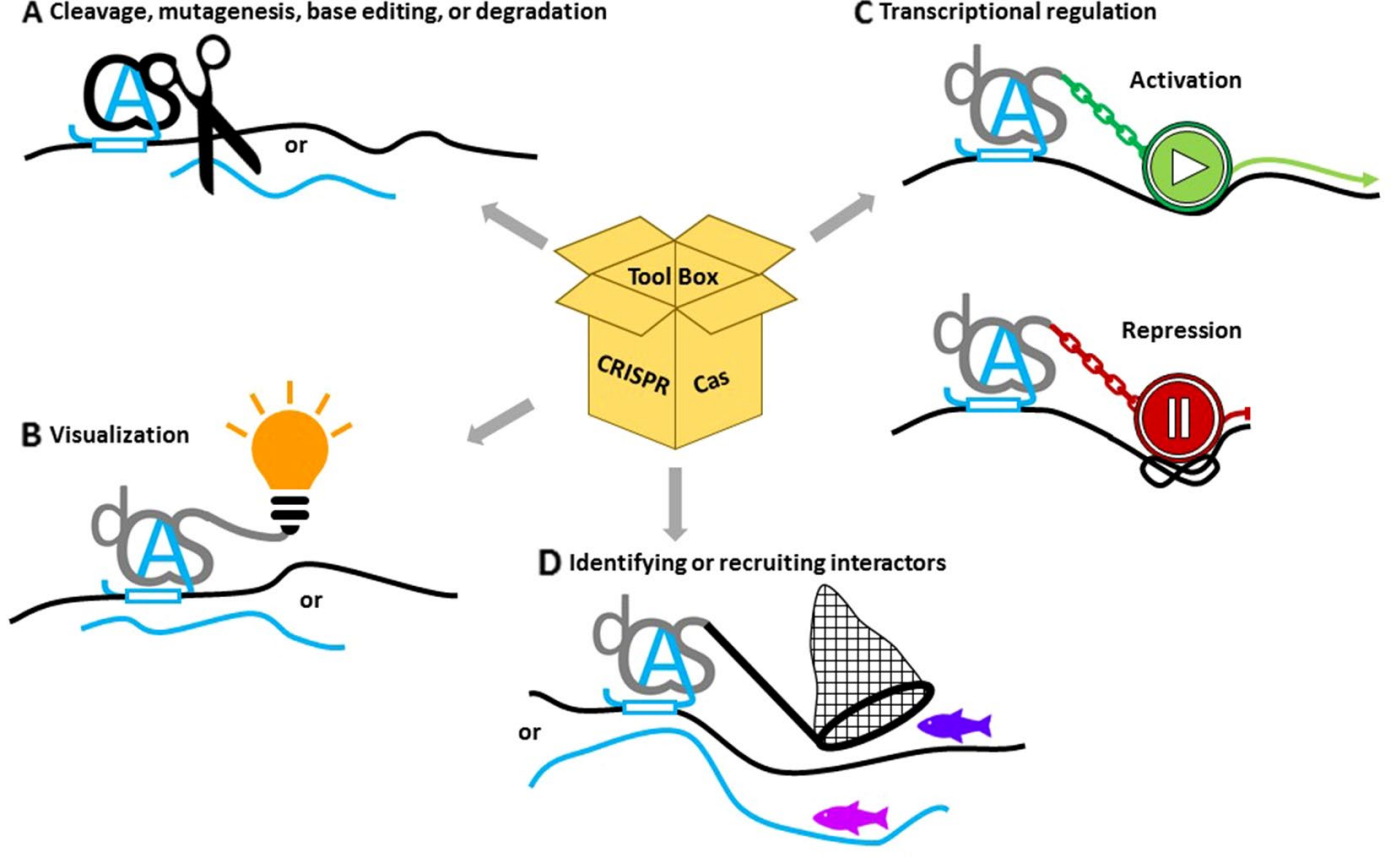
Applications of CRISPR/Cas to study the role of noncoding RNAs. **(A)** Catalytically active Cas proteins (black) together with guide RNAs (blue A) can induce double- or single-strand lesions or base editing on DNA (black lines) or RNA (blue lines). **(B)** Nuclease-dead versions of deadCas proteins (dCas) (gray) fused with fluorescent tags or immuno-epitopes can help to visualize specific targets. **(C)** Fusions with activating or repressing domains or with chromatin-modifying enzymes can change gene expression. **(D)** Coupling interactive proteins to dCas can assemble additional proteins at the binding site, while binding specificity can be exploited to isolate proteins associated with the site.

The diverse capabilities of CRISPR/Cas systems make them attractive components for research on DNA repair in plants (**Figure 2**). In the following section, we will cover the applications related to its endonuclease activity, as well as applications enabled by exploiting the highly specific binding capabilities of inert Cas proteins provided by the sgRNA, like transcriptional control through the fusion of activator/repressor modules, live imaging through the fusion of fluorescent reporter proteins, or DNA- or RNA-binding studies.

### Endonuclease-Related Applications

Several of the previously mentioned aspects of catalytically active CRISPR/Cas applications are especially beneficial when studying the role of ncRNAs for DNA repair in plants. Besides the potential to create DNA lesions as repair “substrates” at specific sites and times, the possibility for targeted mutagenesis is particularly valuable to create mutant alleles in genes encoding ncRNAs. As ncRNAs have no protein coding potential, their biological functions are less likely to be disturbed by small insertions or deletions that would cause frame shifts in protein coding genes. Driving the endonuclease simultaneously to two distinct sites by expressing two gRNAs, with slightly distant targeting site at the same locus, is more likely to cause loss-of-function alleles for ncRNA genes. Such deletions have been routinely produced for several types of genes in plants. For instance, by multiplexing different gRNAs, Zhou et al. (2014) reported large chromosomal deletions in genes involved in sugar efflux transport in rice. Generation of small (<100 bp) and larger (up to 120 kb) deletions were also obtained in *A. thaliana* and *Nicotiana benthamiana* and were stably inherited in the next generation (Ordon et al., 2017; Durr et al., 2018). This suggests that deletion of ncRNA loci can also be easily achieved. By changing the target sites, the deletion size can be controlled and varied, potentially providing information about the role of lncRNA substructures like protein-interacting sites, structurally important domains, or specific regulatory elements. One example is the identification of the A-repeat region of the *XIST* lncRNA that mediates chromosome X inactivation in mammals by interacting with the SPEN transcriptional repressor (Wutz et al., 2002; Lu et al., 2016). However, such approaches require at least some preliminary knowledge about the lncRNA functions.

Generation of lncRNA knockout mutants using CRISPR/Cas9 does have limitations. Many lncRNAs are derived from antisense, bidirectional transcription, or they overlap with protein-coding genes. Indeed, it is hard not to inadvertently affect neighboring genes. A study performed in human cells reveals that only 38% of 15,929 lncRNAs are safely amenable for CRISPR application (Goyal et al., 2017). While this naturally depends on the gene density of the target region and the genome, the growing range of Cas variants might help in some cases to better choose suitable sites. For example, the orthologue Cas12a has higher efficiency for A/T-rich sequences found at many RNA Pol II promoters (Zetsche et al., 2015; Bernabe-Orts et al., 2019). Cas12a-mediated mutagenesis and heritability have been reported in rice and tobacco (Endo et al., 2016; Xu et al., 2017). A higher mutagenesis frequency of biallelic mutations was achieved with a ribozyme-based system, also proving the potential of Cas12 for mutagenesis (Tang et al., 2017). Moreover, multiplexing can be applied as Cas12a has RNase activity and can also simultaneously process its sgRNAs (Wang et al., 2017).

If the genes themselves cannot be mutagenized, their RNA product can be degraded by Cas versions like Cas13. This was demonstrated across a broad range of organisms. In rice, cotransfection with lwaCas13a and sgRNAs into protoplasts leads to a simultaneous reduction of expression of 50% of three genes after 48 h (Abudayyeh et al., 2017). This indicates that Cas13 can rapidly diminish the level of cytoplasmic transcripts. Combined with inducible promoter systems, like AlcR/AlcA (ethanol-inducible), XVE/OlexA (β-estradiol-inducible), pOp/LhGR (dexamethasone-inducible), or with heat shock promoters, all established for use in plants (Borghi, 2010), temporal control of Cas13 expression and consequently of the target RNA level is possible.

Specific RNA degradation *in vivo* by Cas variants in combination with sgRNAs resembles the RNA interference principle (Vaucheret et al., 2001), but as both components are provided by the researcher, the effects are more independent from the presence of endogenous components like Dicer and RISC. This provides great flexibility in experimental conditions and can have considerable advantages for studying ncRNAs in response to DNA damage: (1) direct impact on the RNA level with no need for further plant generations; (2) control of dosage, complementing traditional knockout studies especially when knockouts are lethal; and (3) with a nuclear localization signal, it is also suitable for targeting RNAs during nuclear DNA repair. As *in vitro* analyses revealed some unspecific degradation of RNAs (East-Seletsky et al., 2016), collateral RNA degradation cannot be excluded, but has not been reported in *in vivo* studies in mammals and plants.

### Transcriptional Regulation and CRISPR Display

The DNA-binding specificity of Cas-sgRNA complexes lacking nuclease activity provides valuable possibilities to tether other proteins to the target, and these will also support studies into the role of lncRNAs. In CRISPR interference (CRIPSRi), the protein fused to dCas9 is a repressor domain, which reduces transcription if targeted to a site in the vicinity of a promoter. Repression of both protein- and non–protein-coding genes (miRNAs) has been demonstrated in plants (Lowder et al., 2015; Piatek et al., 2015). By dCas9 and the SRDX transcription repressor domain, the authors achieved a 60% decrease of *AtCSTF64* gene expression, and 50% or more for miRNA159A/miRNA159B (Lowder et al., 2015). This strategy can therefore be applied to inactivate lncRNAs identified to be potential factors in DNA repair.

The same principle is suited for activation of transcription at the target site. The combination of dCas9 with the transcriptional activator domain VP64 (herpes simplex virus protein tetramer repeat sequence) and/or other domains leads to an upregulation of protein-coding and non–protein-coding genes (Lowder et al., 2015; Park et al., 2017), and modification of the guide RNA scaffold improved the transcriptional activation even further (Lowder et al., 2018). Moreover, multiple gRNAs targeting the same promoter achieved a synergistic effect, suggesting that transcription levels can be fine-tuned with this approach (Qi et al., 2013). In addition to modulating transcriptional activity directly, targeting dCas9 can be applied for epigenetic modifications at specific sites, as shown in several studies (Hilton et al., 2015; Papikian et al., 2019). This drives gene activation of genes that usually carry inactive chromatin marks. Such programmable transcription offers the possibility to modulate the expression of ncRNAs without altering the genomic DNA sequence. dCas9 has also been integrated into CRISPR-display, where interaction with a modified sgRNA bearing additional accessory RNA domains outside of the complementary sequence to the target site allows site-specific delivery of larger (>4 kb) aptameric RNAs to specific regions within the genome (Shechner et al., 2015). This can be used to assess if RNA molecules act in *cis* or *trans*, independently of their transcription site, or to tether RNA-binding proteins or ribonucleoprotein complexes. So far only described for mammalian cells, it should be possible to establish CRISPR display in plant systems.

### Live-Imaging

The potential of Cas proteins to tolerate many different fusions without losing interaction with the sgRNA stimulated also several attempts to apply it for live imaging to localize specific sequences within cells. dCas9 protein was coupled with fluorescent tags and used to visualize genomic loci in mammals and plants (Chen et al., 2013; Nelles et al., 2016; Dreissig et al., 2017; Duan et al., 2018; Ishii et al., 2019). While so far mostly repetitive targets gave sufficient signal-to-noise ratios, first attempts with mRNA trafficking (Abudayyeh et al., 2017) are promising and suggest that this system will be adaptable in plants to study ncRNA in response to DNA damage.

### Identification of lncRNA-Associated Proteins

Many RNA-binding proteins play a role in genome stability and DDR signaling. For instance, some are involved in the regulation of RNA/DNA hybrids at the damage site (Li et al., 2016; Cristini et al., 2018; D’Alessandro et al., 2018), others interact with DNA repair factors like BRCA2 or RAD51 (Dutertre et al., 2014). Thus, it is likely that lncRNAs have proteins partners. By fusing an affinity tag such as the polyhistidine (HIS) or glutathione S-transferase molecules to a catalytically inactive Cas13 version with a complementary sgRNA, it will likely be possible to specifically precipitate the targeted lncRNA and collect and analyze proteins bound to it.

## Conclusions and Perspectives

The number of ncRNAs identified to date is larger than our initial expectations, and the list continues to grow rapidly. The roles of ncRNAs in differentiation, development, adaptation, stress response, and inheritance are just beginning to be understood. Long-noncoding RNAs connected with DNA damage are still largely unexplored. The large diversity of applications of the CRISPR toolbox opens new dimensions for genome editing, modification of gene expression or chromatin, or visualization of specific regions, all well documented to work in different plant species. As its application allows the precise and tightly controlled creation of DNA damage, it raises the study of DNA repair directly at the site of the lesion to a new level. This same precision further allows to the generation of targeted mutations in genes encoding for repair factors, including ncRNAs, to study the consequences of loss-of-function mutations. The potential of CRISPR/Cas applications to study ncRNAs is further extended by the diversity of Cas protein variants, especially those targeting RNA molecules. It opens the possibility of degrading RNA molecules as well as base editing, base modification, highly selective screens for interacting components, and subcellular localization. This represents an enormous potential to assist research on ncRNA functions in DNA repair and to extend our knowledge of how ncRNAs participate in the maintenance of genome stability.

## Author Contributions

ND and OS both wrote the review.

## Funding

We appreciate financial support from the Austrian Academy of Sciences to OS and the Austrian Science Fund (M2410) to ND.

## Conflict of Interest

The authors declare that the research was conducted in the absence of any commercial or financial relationships that could be construed as a potential conflict of interest.

## Abbreviations

a-NHEJ, alternative nonhomologous end joining; Cas, CRISPR-associated protein; CRISPR, clustered regularly interspaced short palindromic repeats; CRISPRi, CRISPR interference; dCas, catalytically inactive (dead) Cas; DDR, DNA damage response; DDRNA, DNA damage-response RNA; dilncRNA, damage-induced long-noncoding RNA; diRNA, DSB-induced small RNA; DSB, double-strand break; ɣH2A.X, phosphorylated histone H2A.X; HR, homologous recombination; lncRNA, long noncoding RNA; ncRNA, noncoding RNA; NHEJ, nonhomologous end joining; PAM, protospacer adjacent motif; RDR, RNA-dependent RNA polymerase; RISC, RNA-induced silencing complex; RNAi, RNA interference; sgRNA, single guide RNA; sncRNA, short noncoding RNA.
